# Prognostic Significance of Nodal Burden According to ABO Blood Group in Stage II–III Breast Cancer: An Exploratory Analysis

**DOI:** 10.3390/cancers18213448

**Published:** 2026-10-25

**Authors:** Koji Tezuka, Seiichiro Nishimura, Tomomi Hayashi, Emima Bekku, Yukiko Tadokoro, Rikako Yamamura, Takayoshi Uematsu, Kazuaki Nakashima, Nahomi Tokudome, Takashi Sugino, Makoto Nagahara, Kaoru Takahashi

**Affiliations:** 1 Department of Breast Surgery, JCHO Mishima General Hospital, Mishima 411-0801, Japan; shinjyo_koji@hotmail.com (K.T.);; 2 Department of Breast Surgery, Shizuoka Cancer Center, Nagaizumi 411-8777, Japan; 3 Department of Breast Imaging and Breast Interventional Radiology, Shizuoka Cancer Center, Nagaizumi 411-8777, Japankaz.nakashima@scchr.jp (K.N.); 4 Department of Breast Oncology, Shizuoka Cancer Center, Nagaizumi 411-8777, Japan; 5 Department of Pathology, Shizuoka Cancer Center, Nagaizumi 411-8777, Japan

**Keywords:** ABO blood group, breast cancer, prognosis, lymph node metastasis

## Abstract

Background/Objectives: Previous studies of ABO blood group and breast cancer prognosis have yielded inconsistent results, mainly evaluating blood group itself as a prognostic factor. We investigated whether the prognostic associations of clinicopathological factors differ according to ABO blood group in stage II–III breast cancer. Methods: We retrospectively analyzed 1540 patients with stage II–III breast cancer who underwent curative resection between 2010 and 2019. Blood group-specific prognostic analyses of breast cancer-specific survival (BCSS) were performed, with continuous variables analyzed without dichotomization. Based on the observed patterns, an exploratory interaction between blood group B and pathological nodal status was tested, and its robustness was assessed using a permutation test and sensitivity analyses. Results: ABO blood group was not associated with BCSS overall (*p* = 0.641). In blood group-stratified multivariable analyses, ≥4 lymph node metastases was an independent adverse prognostic factor in blood groups A, O, and AB, but not in blood group B. A significant interaction was observed between blood group B and pathological nodal status (0–3 vs. ≥4 nodes) (*p* for interaction < 0.001; permutation-adjusted *p* = 0.004). Among patients with 0–3 lymph node metastases, blood group B was independently associated with worse BCSS (hazard ratio, 2.04; 95% confidence interval, 1.25–3.32; *p* = 0.004), consistently across sensitivity analyses; this association was more evident among patients who received neoadjuvant chemotherapy. In this subgroup, blood group B was also associated with more distant and visceral recurrence (all *p* < 0.05). Conclusions: The prognostic association of nodal burden in stage II–III breast cancer may differ according to ABO blood group. The exploratory findings in patients with limited nodal involvement suggest that blood group B may identify a subgroup characterized by worse BCSS and increased distant and visceral recurrence. Given the small number of events in blood group B with ≥4 nodes and possible differences according to neoadjuvant chemotherapy, these findings are hypothesis-generating and warrant independent validation.

## Simple Summary

Previous studies of ABO blood group and breast cancer prognosis have yielded inconsistent results, mainly evaluating blood group itself as a prognostic factor. We instead investigated whether established prognostic factors have different associations with survival according to ABO blood group in 1540 patients with stage II–III breast cancer. In blood groups A, O, and AB, extensive nodal involvement was associated with worse breast cancer-specific survival, whereas this pattern was not observed in blood group B. An exploratory analysis showed a significant interaction between blood group B and nodal status. Among patients with 0–3 lymph node metastases, blood group B was associated with worse breast cancer-specific survival and more frequent distant and visceral recurrence. The association with breast cancer-specific survival was consistent across multiple sensitivity analyses. The prognostic significance of nodal burden may therefore vary according to ABO blood group. Validation in independent cohorts is required.

## 1. Introduction

The ABO blood group system, first described by Karl Landsteiner in 1900 [1], is expressed not only on erythrocytes but also on epithelial, endothelial, and cancer cells [2,3]. ABO blood group antigens have been implicated in cardiovascular and thrombotic diseases, infectious diseases, and cancer risk and prognosis [4,5,6,7,8,9].

In women, breast cancer is both the most commonly diagnosed cancer worldwide and the leading cause of cancer-related death [10]. Although prognosis varies according to hormone receptor status and human epidermal growth factor receptor 2 (HER2) expression, patients with stage I breast cancer generally have a favorable prognosis with appropriate treatment [11]. Conversely, patients with stage II–III disease are at increased risk of recurrence, underscoring the need to identify poor prognostic factors and optimize multimodal treatment strategies. Accordingly, many pivotal clinical trials of perioperative systemic therapy have focused on patients with stage II–III or high-risk early breast cancer, with their findings informing current clinical practice [12,13,14,15].

Previous studies investigating the association between ABO blood group and breast cancer prognosis have yielded inconsistent results [16,17,18,19,20,21]. A large prospective cohort study, the Nurses’ Health Study, found no association between ABO blood group and survival [21], whereas other studies reported worse outcomes in patients with non-O blood groups [16], particularly among young patients [19], or with blood groups B and AB [17,18]. Associations between ABO blood group and tumor characteristics have also been examined. Klimant et al. found no significant association between ABO blood group and hormone receptor or HER2 status [20], and in a study restricted to triple-negative breast cancer, Yu et al. found no significant association between ABO blood group and breast cancer-specific mortality [22]. A large nationwide study likewise found no significant association between ABO blood group and estrogen receptor (ER) or HER2 status [23], whereas Stamatakos et al. reported an association between blood group A and ductal breast cancer [24]. Thus, a consistent relationship between ABO blood group and breast cancer phenotype or subtype-specific prognosis has not been established.

Notably, previous prognostic studies have generally examined the association between ABO blood group and survival rather than whether the prognostic significance of established clinicopathological factors differs according to blood group. In other malignancies, however, studies have suggested that the prognostic association of ABO blood group may differ by disease stage [25] and that ABO blood group may modify the prognostic impact of systemic inflammatory markers [26]. Histo-blood group antigens have also been implicated in tumor cell adhesion, motility, and interactions with the vascular endothelium [2,27], raising the possibility that the prognostic significance of established clinicopathological factors may differ according to ABO blood group. If such differences exist, an overall association between ABO blood group and survival could be diluted or masked depending on the clinicopathological composition of each cohort, potentially contributing to the inconsistent findings. However, to our knowledge, whether ABO blood group modifies the prognostic significance of these factors, particularly nodal burden, has not been investigated in breast cancer.

Therefore, in this exploratory study, we investigated whether the prognostic significance of established clinicopathological factors differs according to ABO blood group in patients with stage II–III breast cancer.

## 2. Methods

### 2.1. Patients and Study Design

Between January 2010 and December 2019, 1625 consecutive patients with stage II–III breast cancer underwent surgery at Shizuoka Cancer Center. After excluding patients who received systemic therapy for other malignancies (*n* = 8), had no axillary staging procedure (*n* = 9), had bilateral invasive breast cancer with discordant subtypes (*n* = 24), or had incomplete data (*n* = 44), 1540 patients were included in the analysis.

ABO blood groups were determined preoperatively using concordant forward and reverse typing, and Rh(D) status was also recorded. Staging was defined according to the 8th edition of the Union for International Cancer Control classification [28]. Patients undergoing upfront surgery were included based on pathological stage II–III. Patients receiving neoadjuvant chemotherapy (NAC) were included if they had clinical stage II–III before NAC or pathological stage II–III at surgery. Under these criteria, 271 patients with clinical stage 0–I at diagnosis but pathological stage II–III at surgery were included.

Clinicopathological characteristics, including ABO blood groups, treatment details, and survival outcomes, were obtained from a prospectively maintained database and analyzed retrospectively. Performance status was assessed using the Eastern Cooperative Oncology Group (ECOG) scale. Preoperative evaluation, including staging, tumor extent, and distant metastases, was performed using physical examination, ultrasonography (US), dynamic magnetic resonance imaging (MRI), and positron emission tomography–computed tomography (PET/CT). Lymph node metastasis was primarily assessed via histological or cytological confirmation using core needle biopsy (CNB) or fine-needle aspiration cytology (FNAC). In patients with bilateral breast cancer of the same subtype, the more advanced lesion was analyzed.

### 2.2. Interpretation of Immunohistochemical and Pathological Findings

Immunohistochemical staining for hormone receptors (ER and progesterone [PgR] receptors), HER2, and Ki-67 was performed on breast tumor biopsy and surgical resection specimens, when available. Hormone receptor positivity was defined as nuclear expression of ER or PgR in ≥1% of tumor cells [29]. HER2 status was assessed by immunohistochemistry (IHC) and scored as 0, 1+, 2+, or 3+. HER2 positivity was defined as IHC 3+ or HER2 gene amplification confirmed by fluorescence in situ hybridization (FISH). FISH was performed for equivocal cases (IHC 2+) [30]. Breast cancer subtypes were categorized using hormone receptor and HER2 status, with reference to the general clinical framework of subtype classification [31]. For the present study, tumors were classified as luminal (hormone receptor-positive/HER2-negative), luminal-HER2 (hormone receptor-positive/HER2-positive), HER2 type (hormone receptor-negative/HER2-positive), or triple-negative (hormone receptor-negative/HER2-negative). For subsequent analyses, luminal-HER2 and HER2 types were combined into a single HER2-positive type.

The Ki-67 labeling index was evaluated in hotspot areas of invasive tumor components, with the higher value recorded when both biopsy and surgical specimens were available [32]. Histological grade was assessed using the Elston-Ellis criteria, with the higher grade adopted when both biopsy and surgical specimens were available [33].

### 2.3. Surgical Procedures

Based on preoperative axillary assessment, axillary lymph node dissection (ALND) was performed in patients with clinically positive axillary lymph nodes, whereas sentinel lymph node biopsy using a dual-tracer technique was performed in those with clinically negative axillary lymph nodes. Additional ALND was performed when clinically indicated based on sentinel lymph node findings.

### 2.4. Radiotherapy

Radiotherapy was administered to the remaining breast after breast-conserving surgery. Post-mastectomy radiotherapy (PMRT) to the chest wall and regional lymph nodes was considered for patients with ≥4 pathological lymph node metastases or 1–3 positive nodes with extensive lymphatic invasion [34]. For analytical purposes, patients were categorized into three groups according to radiotherapy status: breast irradiation alone, breast plus regional irradiation, and no irradiation. Breast irradiation alone was defined as irradiation to the conserved breast without regional nodal irradiation. Breast plus regional irradiation included irradiation to the conserved breast with regional nodal irradiation (axillary, supraclavicular, and/or internal mammary), as well as PMRT with regional nodal irradiation after mastectomy. No irradiation was defined as the absence of postoperative radiotherapy.

### 2.5. Perioperative Systemic Therapy

Perioperative systemic therapy was administered according to institutional protocols and contemporaneous guidelines, which evolved over the study period [31,35,36]. In general, chemotherapy for luminal-type breast cancer and triple-negative breast cancer (TNBC) comprised sequential anthracycline-based regimens (epirubicin and cyclophosphamide [EC] or fluorouracil, epirubicin, and cyclophosphamide [FEC] for four cycles), followed by taxane-based regimens (docetaxel for four cycles or paclitaxel for twelve cycles). For HER2-positive breast cancer, anthracycline- and taxane-based regimens were used, with anti-HER2 therapy (trastuzumab with or without pertuzumab) administered concomitantly with taxanes and continued for 1 year, including the preoperative period. NAC was commonly employed for HER2-positive breast cancer and TNBC, as well as for luminal-type breast cancer with a high recurrence risk, typically stage ≥IIB. Depending on the treatment period, selected patients with TNBC who had residual disease after NAC received adjuvant capecitabine.

Chemotherapy regimens and intensity were individualized based on patient factors (age, comorbidities, and performance status) and tumor-related risk factors, including hormone receptor status, Ki-67 index, and other pathological features. Selected patients received de-escalated regimens, such as EC/FEC (four to six cycles) or docetaxel and cyclophosphamide (TC; four cycles).

In patients with luminal-type or luminal-HER2-type breast cancer, adjuvant endocrine therapy was administered for 5–10 years according to recurrence risk. Premenopausal patients were primarily treated with tamoxifen with or without a luteinizing hormone-releasing hormone analogue, whereas postmenopausal patients generally received an aromatase inhibitor. In this study, chemotherapy was defined as the administration of cytotoxic agents, regardless of concomitant targeted therapy. Pathological complete response (pCR) was defined as the complete absence of residual invasive carcinoma in both the breast and axillary lymph nodes (ypT0/is ypN0). For analyses of chemotherapy completion rates, treatment was considered completed if the overall planned treatment intensity was maintained, even when regimen modifications were required.

### 2.6. Follow-Up

After completion of systemic therapy other than endocrine therapy, patients were followed every 3 months for the first 5 years postoperatively and annually thereafter. During follow-up, imaging examinations, including US, mammography, and multidetector computed tomography, were performed annually. Recurrence was diagnosed based on integrated clinical and imaging assessments, including PET/CT, with pathological confirmation by CNB or FNAC when feasible. Recurrence was defined as any local, regional, or distant event, whereas ipsilateral or contralateral breast cancers considered new primary tumors were excluded. Visceral recurrence was defined as recurrence involving the lung, liver, brain, pleura, peritoneum, or other intra-abdominal organs, excluding bone, lymph node, and soft-tissue recurrence.

### 2.7. Statistical Analysis

Continuous variables are presented as medians with interquartile ranges (IQRs) and were compared using the Wilcoxon rank-sum or Kruskal–Wallis test, as appropriate. Categorical variables were compared using the chi-square or Fisher’s exact test.

Prognosis was evaluated using breast cancer-specific survival (BCSS), defined as the time from surgery to death from breast cancer; patients who died of other causes or were alive at the last follow-up were censored. The median follow-up was estimated using the reverse Kaplan–Meier method. The main prognostic analyses aimed to assess whether the prognostic impact of clinicopathological factors differed according to ABO blood group. BCSS curves were estimated using the Kaplan–Meier method and compared using the log-rank test. Age, Ki-67 labeling index, and pathological tumor size were analyzed as continuous variables (per 10 years, per 10%, and per 10 mm, respectively); penalized spline analyses showed no significant departure from linearity (*p* = 0.36, 0.79, and 0.085, respectively). For prognostic factor analyses in the overall cohort, ABO blood group-stratified analyses, and the subgroup with 0–3 pathological lymph node metastases, variables with *p* < 0.05 (log-rank test for categorical variables and likelihood-ratio test for continuous variables) in univariate analyses were included in multivariable Cox proportional hazards models.

To further evaluate whether the prognostic impact of clinicopathological factors—particularly pathological nodal status—differed according to ABO blood group, an exploratory interaction analysis was performed. Pathological nodal status was selected for this analysis based on the distinct prognostic pattern observed in the blood group-specific analyses and the established clinical relevance of the ≥4-node threshold. An interaction term between blood group B (B vs. non-B) and pathological nodal status (0–3 vs. ≥4 metastatic lymph nodes) was included in a multivariable Cox proportional hazards model. In contrast to the prognostic factor analyses described above, the interaction model was adjusted for clinically relevant prognostic factors irrespective of their statistical significance in univariate analyses, including age, clinical stage (III vs. 0–II), breast cancer subtype (luminal vs. non-luminal [luminal-HER2, HER2, and triple-negative]), Ki-67 index, histological grade, pathological tumor size, lymphovascular invasion, neoadjuvant and/or adjuvant chemotherapy, and radiation therapy. Hazard ratios (HRs) with 95% confidence intervals (CIs) were estimated using Cox proportional hazards models, with *p* values for individual covariates based on Wald tests. The statistical significance of the interaction term was evaluated using a likelihood-ratio test comparing nested Cox models with and without the interaction term. Five- and 10-year BCSS rates with 95% CIs were estimated using the Kaplan–Meier method according to blood group and nodal status. The same interaction was also examined for each ABO blood group (each group vs. all others). In addition, blood group-specific HRs for nodal burden (log_2_[number of metastatic nodes + 1]), tumor size, and Ki-67 labeling index were estimated from a single Cox model in the overall cohort that included blood group-specific terms for each factor, and heterogeneity across blood groups was assessed using a likelihood-ratio test.

To assess the robustness of the interaction, several additional analyses were performed. First, because blood group B was selected from among the four ABO blood groups, a permutation test was conducted: ABO blood group labels were randomly permuted 1000 times, the interaction model was fitted for each of the four blood groups in each permuted dataset, and the smallest interaction *p* value was recorded. The permutation-adjusted *p* value was the proportion of permutations in which this minimum was less than or equal to the observed *p* value for blood group B. Second, the internal stability of the interaction estimate was assessed using bootstrap resampling (1000 replicates); resamples in which the model did not converge were excluded. Third, sensitivity analyses included Firth penalized Cox regression to address sparse events, a parsimonious model adjusted only for clinical stage and Ki-67 labeling index, an unadjusted model, chemotherapy categorized as none, adjuvant only, or neoadjuvant, dichotomized covariates using three sets of cutoffs: those derived from standard receiver operating characteristic (ROC) analysis without consideration of follow-up time using the Youden index (age 66 years, Ki-67 labeling index 55%, and tumor size 28 mm), those derived from time-dependent ROC analysis at 5 years with inverse probability of censoring weighting, and conventional cutoffs; and exclusion of Rh-negative patients. The proportional hazards assumption was assessed using Schoenfeld residuals, and covariates violating the assumption were modeled with time-varying coefficients (covariate × log time) in a sensitivity analysis.

To assess the influence of NAC, a three-way interaction (blood group B × nodal status × NAC) was tested in the overall cohort. Among patients with 0–3 lymph node metastases, the interaction between blood group B and NAC was evaluated in a model adjusted for the covariates listed above except chemotherapy. Among patients who received NAC, BCSS was further examined according to residual nodal status (ypN0 vs. ypN1–3) and clinical stage.

Cumulative incidence of recurrence was compared using Gray’s test, treating death without recurrence as a competing risk.

For the number of pathological lymph node metastases, a cutoff of ≥4 was employed rather than a data-driven threshold, based on its established clinical relevance in distinguishing pN1 from pN2 in TNM staging [28] and its widespread use in guiding treatment decisions, such as post-mastectomy radiation therapy [34].

All statistical analyses were performed using JMP (version 14.2; SAS Institute Inc., Cary, NC, USA), EZR (version 1.68; Jichi Medical University, Saitama, Japan), or R (version 4.3.3; R Foundation for Statistical Computing, Vienna, Austria) with the survival package (version 3.5-8). A *p* value < 0.05 was considered statistically significant. Because this was an exploratory study, corrections for multiple testing were not applied, except for the permutation-based adjustment of the interaction test.

## 3. Results

### 3.1. Patient Characteristics and Prognosis According to ABO Blood Group

Patient characteristics are summarized in Table 1. The median follow-up, estimated using the reverse Kaplan–Meier method, was 106.8 months (IQR, 77.5–120.3), and 1540 patients were included. ABO blood group distribution was: A, 38.6%; O, 30.1%; B, 21.8%; and AB, 9.5%. Thirteen patients (0.8%) were Rh-negative. Baseline clinicopathological characteristics were comparable across the patients with different blood groups. No significant differences in BCSS were observed according to ABO blood group in univariate analysis (*p* = 0.641) (Figure S1) or in multivariable analysis (overall *p* = 0.283) (Table S1).

### 3.2. Prognostic Factor Analysis According to ABO Blood Groups

Univariate and multivariate analyses of BCSS in the overall stage II–III cohort and those stratified by ABO blood group are presented in Table S1 and Table 2, respectively.

**Figure 1:**
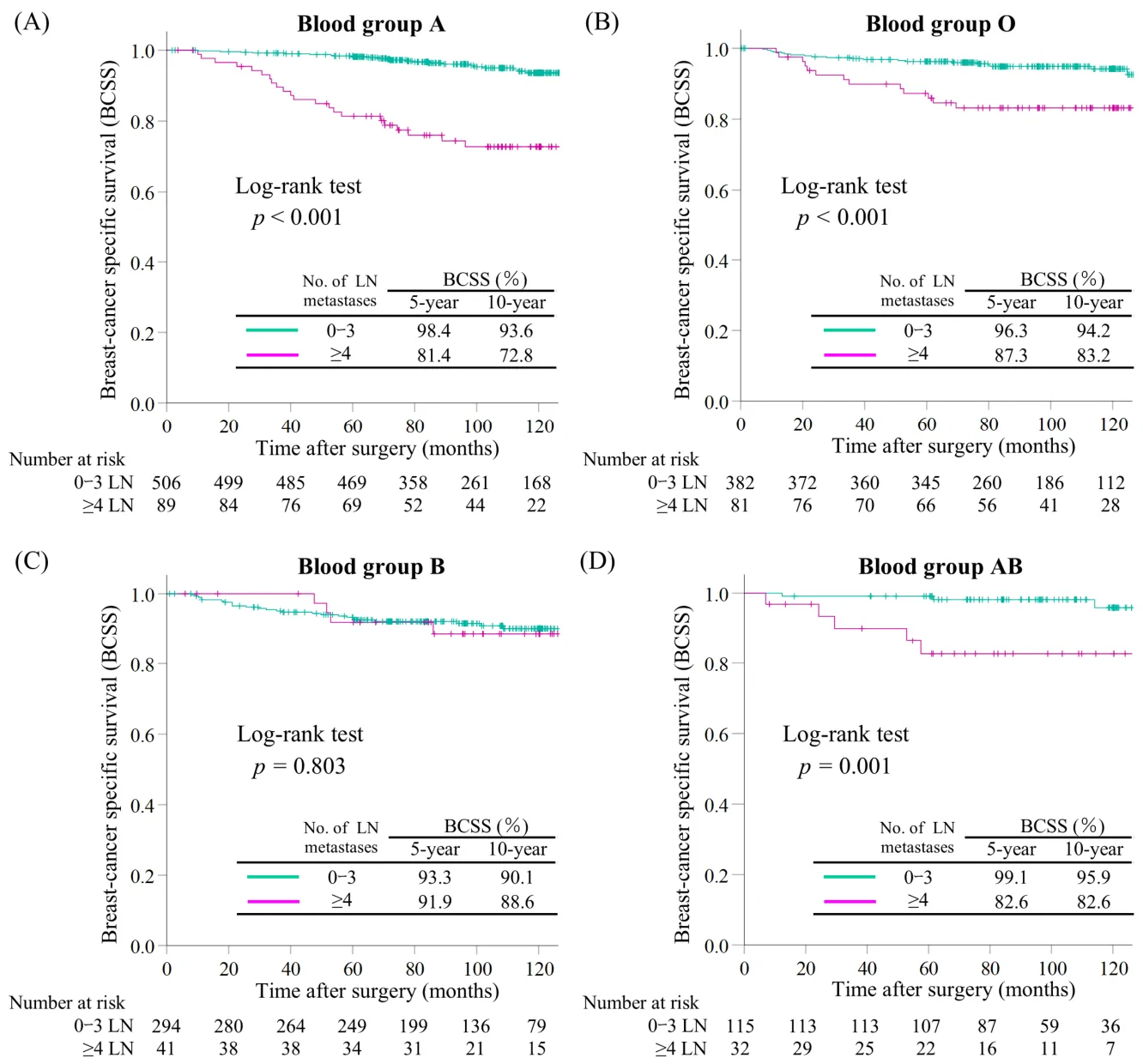
Breast cancer-specific survival (BCSS) stratified by the number of pathological lymph node (LN) metastases (0–3 vs. ≥4) according to ABO blood group in patients with stage II–III breast cancer. Kaplan–Meier curves for BCSS are shown for blood groups A (**A**), O (**B**), B (**C**), and AB (**D**). Survival differences between patients with 0–3 and ≥4 pathological LN metastases were compared using the log-rank test. A significantly poorer prognosis associated with ≥4 pathological LN metastases was observed in blood groups A, O, and AB, whereas this association was not observed in blood group B. Numbers at risk are shown below the curves.

**Figure 2:**
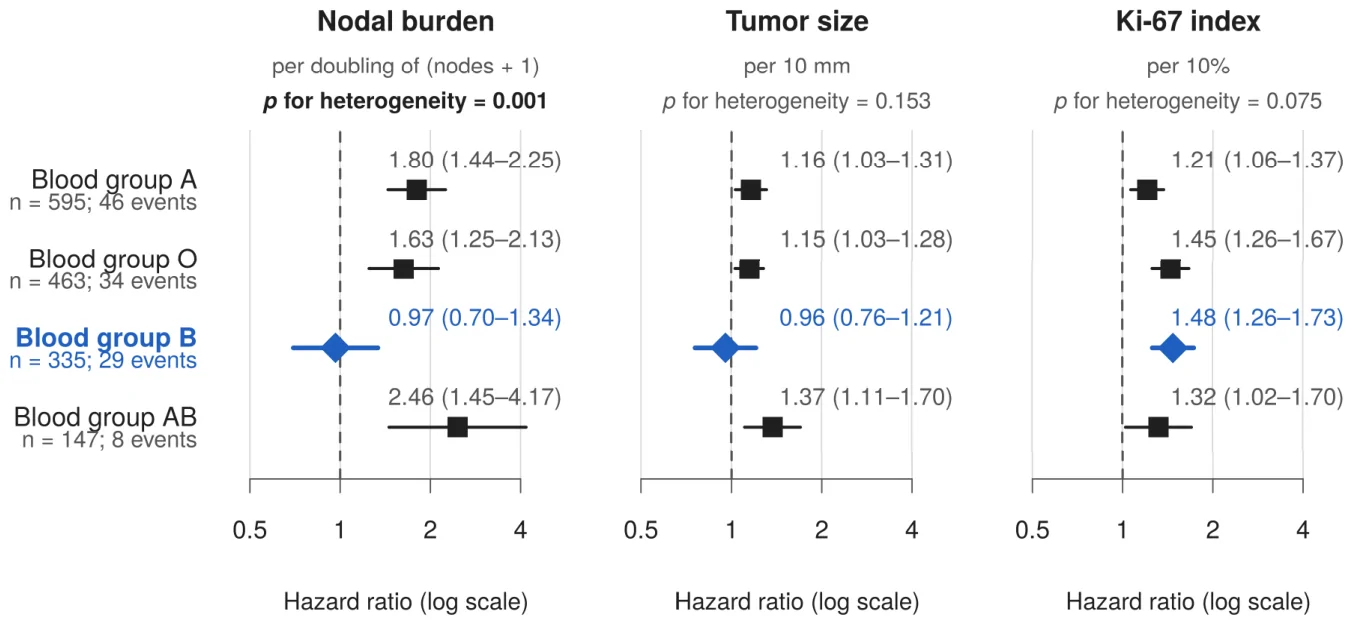
Adjusted hazard ratios for breast cancer-specific survival associated with continuous prognostic factors, by ABO blood group. Blood group-specific hazard ratios were estimated from a single Cox model in the overall cohort that included blood group, blood group-specific terms for the factor of interest, and the remaining covariates (age, clinical stage III, non-luminal subtype, Ki-67 labeling index, histological grade III, tumor size, ≥4 pathological lymph node metastases, lymphovascular invasion, chemotherapy, and radiation therapy). Nodal burden was modeled as log_2_(number of metastatic nodes + 1). *p* for heterogeneity was obtained by likelihood-ratio test. Squares (diamonds for blood group B) indicate hazard ratios, and horizontal lines indicate 95% confidence intervals; the dashed vertical line indicates a hazard ratio of 1. Abbreviations: BCSS, breast cancer-specific survival.

In the overall cohort, clinical stage III, Ki-67 labeling index, pathological tumor size, ≥4 pathological lymph node metastases, and lymphovascular invasion were independent adverse prognostic factors for BCSS. Stratified analyses revealed distinct prognostic patterns according to ABO blood group. Notably, ≥4 pathological lymph node metastases was an independent adverse prognostic factor in blood groups A, O, and AB, but not in blood group B (Table 2). The corresponding survival curves are shown in Figure 1. In blood group B, the independent adverse prognostic factors were limited to clinical stage III and Ki-67 labeling index (Table 2). Consistently, when nodal burden was modeled as a continuous variable (log_2_[number of metastatic nodes + 1]) with blood group-specific terms in a single multivariable model, the HR for nodal burden was close to 1.0 only in blood group B (HR, 0.97; 95% CI, 0.70–1.34), and the heterogeneity across blood groups was significant (*p* = 0.001). A similar but nonsignificant pattern was observed for tumor size (HR in blood group B, 0.96; *p* for heterogeneity = 0.153), whereas the HR for the Ki-67 labeling index was, if anything, highest in blood group B (1.48; *p* for heterogeneity = 0.075; Figure 2). Similar results were obtained when blood group B was compared with non-B blood groups (Table S3).

### 3.3. Interaction Between Blood Group B and Pathological Nodal Status

**Figure 3:**
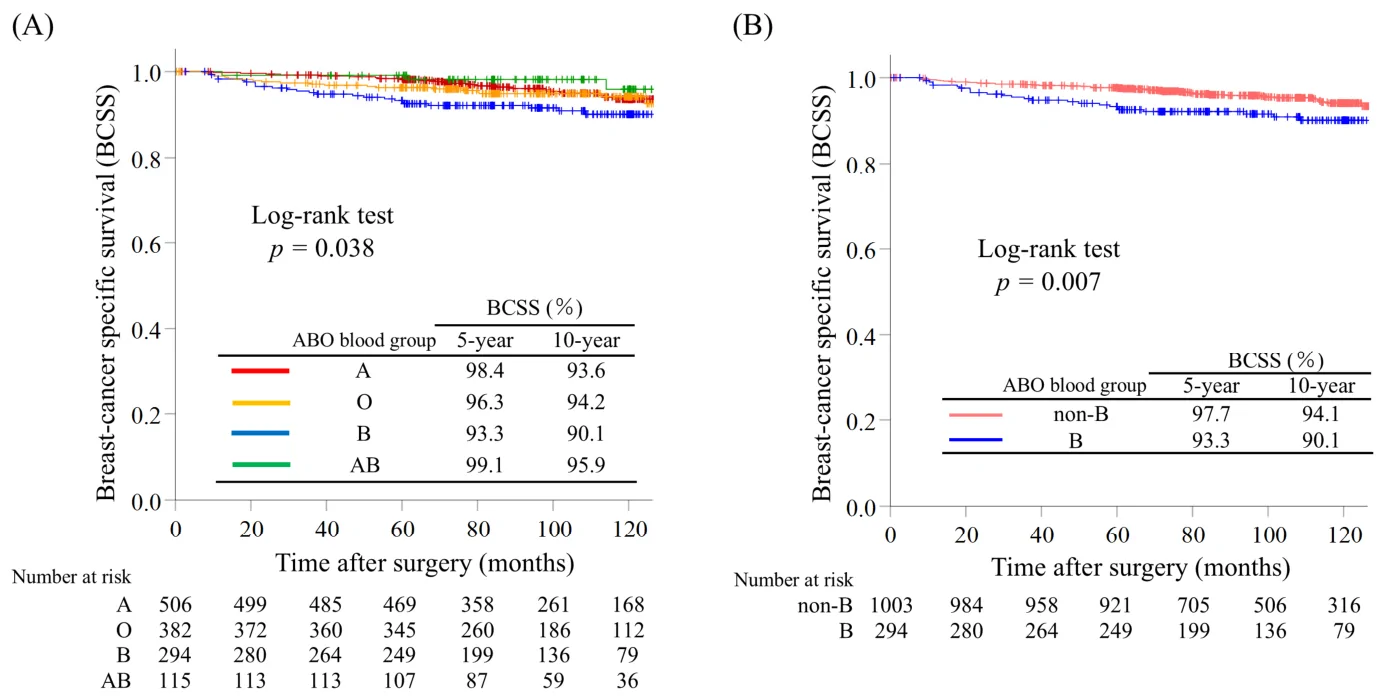
Breast cancer-specific survival (BCSS) according to ABO blood group and comparison between blood group B and non-B blood groups in patients with stage II–III breast cancer with 0–3 lymph node metastases. Kaplan–Meier curves for BCSS are shown according to ABO blood group (A, O, B, and AB) (**A**). BCSS was additionally compared between patients with blood group B and those with non-B blood groups (**B**). Survival differences were assessed using the log-rank test. Numbers at risk are shown below the curves.

To determine whether the prognostic association of blood group B differed according to nodal burden, an interaction analysis was performed. In the multivariable Cox model including the interaction term, the association between blood group B and BCSS differed significantly according to pathological nodal status (*p* for interaction < 0.001; Table 3). Blood group B was associated with significantly worse BCSS among patients with 0–3 lymph node metastases (adjusted HR, 2.00; 95% CI, 1.23–3.26; *p* = 0.005), whereas an adjusted HR of 0.36 (95% CI, 0.13–1.00; *p* = 0.051) was observed among those with ≥4 lymph node metastases; however, this estimate was based on only four events and should be regarded as hypothesis-generating. In patients with 0–3 lymph node metastases, the 5- and 10-year BCSS rates were 93.3% and 90.1% for blood group B and 97.7% and 94.1% for non-B patients, respectively (Table 3). Consistent with this pattern, unadjusted analyses showed significantly poorer BCSS for blood group B in the 0–3 node subgroup (*p* = 0.007; Figure 3), with no significant difference observed in the ≥4 node subgroup (*p* = 0.124; Figure S2).

### 3.4. Sensitivity Analyses and Bootstrap Resampling

All prespecified and post hoc analyses performed in this study are listed in Table S2. Several analyses were performed to assess the robustness of the interaction. First, because blood group B was selected from among the four ABO blood groups, a permutation test (1000 permutations) was used to account for this multiplicity; the permutation-adjusted *p* for interaction was 0.004 (Table 3). When the same interaction was examined for each blood group, a significant interaction was observed for blood group B, an interaction in the opposite direction for blood group A (*p* = 0.015), and none for blood groups O and AB (*p* = 0.85 and 0.20, respectively; Table S3). Second, bootstrap resampling (1000 replicates) to assess internal stability yielded a median adjusted HR of 2.03 (2.5th–97.5th percentiles, 1.18–3.22) for blood group B among patients with 0–3 lymph node metastases, and the interaction was significant in 92.6% of the 989 converged replicates. Third, the interaction remained significant with Firth penalized Cox regression, a parsimonious model, an unadjusted model, alternative categorizations of covariates, chemotherapy categorized as none, adjuvant, or neoadjuvant, exclusion of Rh-negative patients, and time-varying coefficients for covariates violating the proportional hazards assumption (Table S4). The proportional hazards assumption was satisfied for blood group B, nodal status, and their interaction term (Table S3).

### 3.5. Exploratory Analyses in Patients with 0–3 Lymph Node Metastases

Based on this significant interaction, further exploratory analyses were conducted in patients with 0–3 lymph node metastases to characterize this subgroup with limited nodal involvement. In multivariable analysis restricted to this cohort, blood group B remained independently associated with worse BCSS (adjusted HR, 2.04; 95% CI, 1.25–3.32; *p* = 0.004), along with clinical stage III, Ki-67 index, pathological tumor size, and lymphovascular invasion (Table S5). In this subgroup, the proportion of patients receiving NAC (40.8% vs. 39.3%; *p* = 0.636) and pCR rates (29.2% vs. 26.9%; *p* = 0.641) were comparable between blood group B and non-B patients (Table S6).

Notably, despite their poorer survival, patients with blood group B had a significantly lower frequency of ≥2 lymph node metastases than non-B patients (14.3% vs. 19.8%; *p* = 0.033; Table S6). Exploratory recurrence analyses further demonstrated significantly higher cumulative incidences of overall, distant, visceral, and multiple-organ recurrence in blood group B than in non-B patients (all *p* < 0.05, Gray’s test; Figure S3).

Because pathological nodal status may be affected by NAC, the influence of NAC was further examined. No significant three-way interaction was observed between blood group B, nodal status, and NAC status (*p* for three-way interaction = 0.553; Table S7). Among patients with 0–3 lymph node metastases, however, the adverse association of blood group B was observed in those who received NAC (adjusted HR, 3.00; 95% CI, 1.63–5.53; *p* < 0.001) but not in those who underwent upfront surgery (adjusted HR, 0.97; 95% CI, 0.38–2.46; *p* = 0.942; *p* for interaction = 0.024; Figure S4 and Table S7). Among NAC-treated patients, the higher breast cancer mortality in blood group B was observed in both ypN0 (12.8% vs. 3.8%) and ypN1–3 disease (21.4% vs. 13.0%; *p* for interaction = 0.33), as well as in both clinical stage 0–II (12.3% vs. 5.4%) and III disease (23.1% vs. 10.5%; *p* for interaction = 0.70; Table S7). Among patients who underwent upfront surgery with ≥4 lymph node metastases, breast cancer deaths were less frequent in blood group B than in non-B patients (4.8% vs. 13.3%; Table S7).

## 4. Discussion

This exploratory analysis suggests that the prognostic impact of established clinicopathological factors in stage II–III breast cancer may vary according to ABO blood group. A significant interaction was observed between blood group B and pathological nodal status, indicating that the prognostic association of blood group B with BCSS differed according to nodal burden. Among patients with 0–3 lymph node metastases, blood group B was associated with significantly worse BCSS and higher recurrence risk.

Studies have reported inconsistent associations between ABO blood group and breast cancer prognosis [16,17,18,19,20,21,37]. Many investigations found no significant relationship between blood group and breast cancer outcomes [20,21,37], consistent with the results of our overall analysis. However, some studies have reported that blood group B is associated with worse prognosis either together with other non-O blood groups [16,17,19] or alone [18], or with a trend toward worse prognosis [20]. These findings partially support our observation of poorer outcomes for blood group B among patients with 0–3 lymph node metastases. The significant interaction observed in our study may provide one possible explanation for the inconsistent findings of previous studies, as associations between ABO blood group and prognosis could vary according to the distribution of nodal burden within the study population.

Generally, the presence of ≥4 metastatic lymph nodes is an established adverse prognostic factor in breast cancer [28]. However, in the present study, conventional lymph node-based prognostic stratification was not observed in blood group B, with patients demonstrating relatively poor outcomes even when nodal involvement was limited to 0–3 nodes. Notably, despite significantly higher rates of distant and visceral recurrence in this subgroup, patients with blood group B had a lower frequency of multiple lymph node metastases than non-B patients (exploratory comparison; *p* = 0.033). This apparently discordant pattern between nodal burden and systemic recurrence may not be fully explained by a conventional stepwise model of lymphatic progression [38,39]. One possible explanation is that biological heterogeneity exists within blood group B breast cancer, with some tumors having a greater propensity for systemic dissemination despite limited regional nodal involvement. Previous studies have demonstrated organ-specific metastatic patterns in breast cancer and identified molecular characteristics associated with preferential metastatic sites, supporting the concept that metastatic dissemination may reflect underlying tumor biology [40]. Such heterogeneity could contribute to the lack of a clear association between heavy nodal burden and worse BCSS observed in blood group B. However, the present study did not directly investigate mechanisms of metastatic dissemination, and this hypothesis requires biological validation.

The biological mechanisms underlying this apparently discordant pattern remain unclear. However, prior studies have reported associations between the ABO locus and circulating adhesion molecules such as soluble E-selectin, P-selectin, and ICAM-1 [41,42,43], which play important roles in endothelial interactions and metastatic processes [38,44]. In addition, reduced or lost expression of ABO-related histo-blood group antigens in breast carcinoma tissues relative to normal breast epithelium has been reported and may be associated with adverse clinicopathological features and prognosis [3,45]. Patterns of alteration in blood group antigen expression may also differ according to ABO blood group [45]. More broadly, altered expression of ABH and Lewis histo-blood group antigens has been implicated in tumor progression, with loss of A and B antigens associated with increased cellular motility and sialyl-Lewis antigens facilitating interactions with vascular endothelium during the metastatic process [2]. Although these observations raise the possibility that ABO-related biological differences may contribute to the metastatic pattern observed in patients with blood group B, any such relationship remains speculative and requires further biological and clinical investigation.

Clinically, breast cancer with 0–3 lymph node metastases represents a prognostically heterogeneous population. In the present study, pathological tumor size—an established prognostic factor alongside nodal status [28]—was not significantly associated with BCSS in blood group B. Together with the distinct association between nodal burden and prognosis observed in this group, this finding raises the possibility that conventional anatomical risk factors may not fully capture prognostic heterogeneity among patients with blood group B. Further validation is required before these findings can be incorporated into clinical risk stratification.

Because NAC can downstage axillary nodal status, its potential influence on our findings should be considered. Among patients with 0–3 lymph node metastases, the proportion receiving NAC and the pCR rate were comparable between blood group B and non-B patients (Table S6), suggesting that differences in these treatment-related factors alone may not explain our findings. When stratified by treatment sequence, however, the adverse prognosis of blood group B among patients with 0–3 lymph node metastases was observed mainly in those who received NAC and was not evident in those who underwent upfront surgery (Figure S4). Particularly in luminal breast cancer, NAC is generally reserved for patients at high risk of recurrence; NAC-treated patients therefore represent a population with a high baseline risk of systemic relapse, in which prognostic differences may become more apparent. Conversely, among patients who underwent upfront surgery with ≥4 lymph node metastases, breast cancer deaths were less frequent in blood group B than in non-B patients (Table S7). Taken together, these observations are consistent with the notion that nodal burden may less accurately reflect the risk of systemic dissemination in blood group B: extensive nodal involvement may not necessarily be accompanied by systemic spread, and nodal clearance after NAC may not necessarily indicate control of systemic micrometastases. Indeed, even among patients without residual nodal metastases after NAC, breast cancer deaths were more frequent in blood group B than in non-B patients (Table S7). This pattern is in line with the hypothesis that hematogenous dissemination, independent of stepwise lymphatic progression, may play a greater role in blood group B. However, these stratified analyses were based on small numbers of events, and the three-way interaction was not significant; they should therefore be regarded as hypothesis-generating.

The ABO blood group distribution in our cohort (A, 38.6%; O, 30.1%; B, 21.8%; AB, 9.5%) closely mirrored that of the general Japanese population [46], suggesting no substantial selection bias with respect to ABO blood group distribution.

This study has several limitations. First, as a single-center retrospective analysis, selection bias and unmeasured confounding cannot be excluded. Second, the blood group-specific and subgroup analyses were exploratory, and apart from the permutation-adjusted interaction test, no adjustment for multiple testing was applied. In particular, the interaction analysis between blood group B and pathological nodal status was conducted based on findings from the blood group-specific prognostic analyses rather than being prespecified. Although the significant interaction supports the possibility of heterogeneity in the prognostic impact of nodal burden between blood group B and non-B patients, these findings should be considered hypothesis-generating rather than confirmatory. The permutation test accounted only for the selection of blood group B from among the four ABO blood groups and did not account for the other exploratory comparisons performed in this study. Similarly, the bootstrap analysis indicates the internal stability of the estimate within this cohort rather than independent confirmation. In addition, within the ≥4-node stratum, the point estimate for blood group B was in the opposite direction (better BCSS); however, this was based on only four breast cancer deaths among patients with blood group B and is subject to considerable uncertainty and should be interpreted with caution. The adverse prognostic association of blood group B observed in this study should therefore be interpreted primarily in the context of patients with limited nodal involvement, rather than as evidence of a protective effect in those with heavy nodal burden. Likewise, the absence of a significant association between nodal burden and BCSS within blood group B should not be interpreted as evidence that nodal burden lacks prognostic relevance in these patients. Third, the findings were not validated in an independent cohort and require external validation. Fourth, the study population exclusively comprised Japanese patients, and potential ethnic differences in ABO blood group distribution or tumor biology may limit the generalizability of the findings to other populations. Fifth, systemic therapies for breast cancer evolved considerably both during and after the study period (2010–2019). Although perioperative treatments were administered according to contemporaneous guidelines, changes in perioperative treatment strategies over the study period, as well as subsequent advances in post-recurrence therapies during long-term follow-up, may have introduced temporal confounding and influenced BCSS. Finally, the relatively limited sample size, particularly within individual ABO blood groups, limited the precision of the analyses stratified by treatment sequence and precluded detailed analyses according to clinicopathological subtype.

## 5. Conclusions

In conclusion, our exploratory analyses suggest that the prognostic impact of blood group B in stage II–III breast cancer may differ according to nodal burden, as supported by a significant interaction between blood group B and pathological nodal status. While no overall prognostic differences were observed among ABO blood groups, blood group B was associated with significantly worse breast cancer-specific survival among patients with 0–3 pathological lymph node metastases. Thus, ABO blood group may modify the prognostic significance of nodal burden, potentially contributing to the inconsistent findings of previous studies examining the association between ABO blood group and breast cancer prognosis. These findings raise the possibility that underlying biological differences associated with blood group B may contribute to this distinct prognostic pattern; however, they are hypothesis-generating and require independent validation. This need is underscored by the small number of events among patients with blood group B and ≥4 lymph node metastases and by the possibility that the association differs according to neoadjuvant chemotherapy. These findings are not sufficiently validated to guide treatment decisions. Validation in well-designed, large-scale multicenter studies is warranted, and translational studies are needed to elucidate the underlying biological mechanisms.

## Figures and Tables

**Table 1:** Clinicopathological characteristics of patients with stage II–III breast cancer according to ABO blood group.

Variables	All Patients (*n* = 1540)	Blood Group				*p* Value
		A (*n* = 595)	O (*n* = 463)	B (*n* = 335)	AB (*n* = 147)	
Age (years), median (IQR)	57 (47–68)	57 (47–68)	58 (48–68)	57 (46–67)	58 (45–67)	0.501
Gender (Female)	1535 (99.7)	591 (99.3)	463 (100)	334 (99.7)	147 (100)	0.320
Performance status (ECOG ≥ 1)	138 (9.0)	45 (7.6)	44 (9.5)	37 (11.0)	12 (8.2)	0.321
Rh-negative	13 (0.8)	7 (1.2)	5 (1.1)	1 (0.3)	0	0.394
Clinical T stage						0.094
cTis	23 (1.5)	11 (1.8)	6 (1.3)	6 (1.8)	0	
cT1	395 (25.6)	141 (23.7)	123 (26.6)	87 (26.0)	44 (29.9)	
cT2	918 (59.6)	374 (62.9)	272 (58.7)	200 (59.7)	72 (49.0)	
cT3	101 (6.6)	38 (6.4)	29 (6.3)	18 (5.4)	16 (10.9)	
cT4	103 (6.7)	31 (5.2)	33 (7.1)	24 (7.2)	15 (10.2)	
Clinical lymph node status						0.763
cN0	860 (55.8)	341 (57.3)	247 (53.3)	188 (56.1)	84 (57.1)	
cN1	538 (34.9)	206 (34.6)	171 (36.9)	114 (34.0)	47 (32.0)	
cN2–3	142 (9.2)	48 (8.1)	45 (9.7)	33 (9.9)	16 (10.9)	
Clinical stage at diagnosis ^a^						0.071
0	23 (1.5)	11 (1.8)	6 (1.3)	6 (1.8)	0	
I	248 (16.1)	89 (15.0)	73 (15.8)	56 (16.7)	30 (20.4)	
II	993 (64.5)	404 (67.9)	295 (63.7)	214 (63.9)	80 (54.4)	
III	276 (17.9)	91 (15.3)	89 (19.2)	59 (17.6)	37 (25.2)	
Subtypes						0.230
Luminal	979 (63.6)	379 (63.7)	293 (63.3)	210 (62.7)	97 (66.0)	
Luminal-HER2	186 (12.1)	79 (13.3)	53 (11.4)	39 (11.6)	15 (10.2)	
HER2	130 (8.4)	56 (9.4)	28 (6.0)	34 (10.1)	12 (8.2)	
Triple negative	245 (15.9)	81 (13.6)	89 (19.2)	52 (15.5)	23 (15.6)	
Ki-67 labeling index (%), median (IQR)	30 (15–60)	29 (15–59)	30 (15–60)	31 (17–63)	27 (15–61)	0.426
Histological grade						0.506
Grade I	233 (15.1)	84 (14.1)	75 (16.2)	53 (15.8)	21 (14.3)	
Grade II	754 (49.0)	308 (51.8)	224 (48.4)	158 (47.2)	64 (43.5)	
Grade III	553 (35.9)	203 (34.1)	164 (35.4)	124 (37.0)	62 (42.2)	
Histological type						0.713
Invasive ductal carcinoma	1305 (84.7)	503 (84.5)	390 (84.2)	286 (85.4)	126 (85.7)	
Invasive lobular carcinoma	123 (8.0)	51 (8.6)	32 (6.9)	27 (8.1)	13 (8.8)	
Others	112 (7.3)	41 (6.9)	41 (8.9)	22 (6.6)	8 (5.4)	
Pathological tumor size (mm), median (IQR)	21 (11–28)	21 (11–28)	21 (10–28)	20 (10–28)	21 (13–29)	0.450
Number of pathological lymph node metastases						0.111
0	653 (42.4)	256 (43.0)	190 (41.0)	155 (46.3)	52 (35.4)	
1–3	644 (41.8)	250 (42.0)	192 (41.5)	139 (41.5)	63 (42.9)	
≥4	243 (15.8)	89 (15.0)	81 (17.5)	41 (12.2)	32 (21.8)	
Lymphovascular invasion	704 (45.7)	273 (45.9)	206 (44.5)	151 (45.1)	74 (50.3)	0.658
Pathological complete response rate (%) ^b^	22.4 (141/631)	20.9 (49/235)	23.1 (46/199)	25.0 (35/140)	19.3 (11/57)	0.743
Surgical procedure						0.238
Mastectomy	1030 (66.9)	392 (65.9)	299 (64.6)	238 (71.0)	101 (68.7)	
Breast-conserving surgery	510 (33.1)	203 (34.1)	164 (35.4)	97 (29.0)	46 (31.3)	
Axillary surgery						0.750
Sentinel lymph node biopsy only	546 (35.5)	215 (36.1)	156 (33.7)	119 (35.5)	56 (38.1)	
Axillary lymph node dissection	994 (64.5)	380 (63.9)	307 (66.3)	216 (64.5)	91 (61.9)	
Radiation therapy						0.122
Breast irradiation alone	424 (27.5)	164 (27.6)	140 (30.2)	81 (24.2)	39 (26.5)	
Breast and regional irradiation ^c^	412 (26.8)	156 (26.2)	127 (27.4)	81 (24.2)	48 (32.7)	
No irradiation	704 (45.7)	275 (46.2)	196 (42.3)	173 (51.6)	60 (40.8)	
Endocrine therapy	1109 (72.0)	433 (72.8)	328 (70.8)	240 (71.6)	108 (73.5)	0.885
Anti-HER2 therapy	306 (19.9)	135 (22.7)	78 (16.8)	66 (19.7)	27 (18.4)	0.122
Neoadjuvant chemotherapy	631 (41.0)	235 (39.5)	199 (43.0)	140 (41.8)	57 (38.8)	0.640
Adjuvant chemotherapy	511 (33.2)	202 (34.0)	134 (28.9)	117 (34.9)	58 (39.5)	0.070
Chemotherapy regimen						0.338
Anthracycline- and taxane-containing regimens	840 (54.5)	314 (52.8)	255 (55.1)	185 (55.2)	86 (58.5)	
Taxane-containing regimens without anthracycline	124 (8.0)	52 (8.7)	24 (5.2)	35 (10.4)	13 (8.9)	
Anthracycline-containing regimens without taxane	118 (7.7)	48 (8.1)	41 (8.9)	20 (6.0)	9 (6.1)	
Others	9 (0.6)	3 (0.5)	4 (0.9)	2 (0.6)	0	
No chemotherapy	449 (29.2)	178 (29.9)	139 (30.0)	93 (27.8)	39 (26.5)	
Completion rate of intravenous chemotherapy (%) ^d^	90.0 (976/1084)	91.1 (379/416)	90.6 (290/320)	87.5 (210/240)	89.8 (97/108)	0.497

Values are expressed as medians (interquartile ranges) or *n* (%). Percentages are calculated based on the number of patients in each group unless otherwise specified. *p* values represent comparisons among the four ABO blood groups and were calculated using the Kruskal–Wallis test for continuous variables and Fisher’s exact test or the chi-square test for categorical variables, as appropriate. ^a^ Patients with clinical stage 0–I were included based on pathological stage II–III at surgery. ^b^ Pathological complete response (pCR) was defined as ypT0/is ypN0 and was assessed among patients who received neoadjuvant chemotherapy (NAC) (*n* = 631). Values are presented as percentages, with the number of patients achieving pCR/number of patients receiving NAC shown in parentheses. ^c^ Breast and regional irradiation includes postmastectomy radiation therapy (PMRT). ^d^ The completion rate of intravenous chemotherapy was evaluated in 1084 patients who received the treatment. Values are presented as percentages, with the number of patients completing intravenous chemotherapy/number of patients receiving intravenous chemotherapy shown in parentheses. Abbreviations: IQR, interquartile range; ECOG, Eastern Cooperative Oncology Group; HER2, human epidermal growth factor receptor 2.

**Table 2:** Univariate and multivariate analyses of prognostic factors for breast cancer-specific survival according to ABO blood group.

**Variables**	**Blood Group A (*n* = 595; 46 Events)**				**Blood Group O (*n* = 463; 34 Events)**			
	***n* (%)**	**Univariate ^a^**	**Multivariate ^b^**		***n* (%)**	**Univariate ^a^**	**Multivariate ^b^**	
		***p* Value**	**HR (95% CI)**	***p* Value**		***p* Value**	**HR (95% CI)**	***p* Value**
Age (per 10 years), median (IQR)	57 (47–68)	0.985	–	–	58 (48–68)	0.641	–	–
Clinical stage III	91 (15.3)	<0.001	2.32 (1.18–4.56)	0.015	89 (19.2)	0.046	1.16 (0.53–2.57)	0.707
Non-luminal subtypes	216 (36.3)	0.837	–	–	170 (36.7)	0.030	1.31 (0.56–3.08)	0.539
Ki-67 labeling index (per 10%), median (IQR)	29 (15–59)	0.113	–	–	30 (15–60)	<0.001	1.42 (1.17–1.72)	<0.001
Histological grade III	203 (34.1)	0.142	–	–	164 (35.4)	0.043	0.68 (0.27–1.75)	0.428
Pathological tumor size (per 10 mm), median (IQR)	21 (11–28)	0.003	1.04 (0.91–1.19)	0.543	21 (10–28)	0.002	1.19 (1.08–1.32)	<0.001
Number of pathological lymph node metastases ≥4	89 (15.0)	<0.001	3.98 (1.91–8.30)	<0.001	81 (17.5)	<0.001	4.30 (1.92–9.64)	<0.001
Lymphovascular invasion	273 (45.9)	<0.001	2.75 (1.37–5.54)	0.005	206 (44.5)	0.088	–	–
Neoadjuvant and/or adjuvant chemotherapy	417 (70.1)	0.015	1.80 (0.73–4.44)	0.199	324 (70.0)	0.021	1.08 (0.35–3.29)	0.895
Radiation therapy	320 (53.8)	0.011	0.84 (0.38–1.90)	0.684	267 (57.7)	0.265	–	–
**Variables**	**Blood Group B (*n* = 335; 29 Events** **)**				**Blood Group AB (*n* = 147; 8 Events) ^c^**			
	***n* (%)**	**Univariate ^a^**	**Multivariate ^b^**		***n* (%)**	**Univariate ^a^**	**Multivariate ^b^**	
		***p* Value**	**HR (95% CI)**	***p* Value**		***p* Value**	**HR (95% CI)**	***p* Value**
Age (per 10 years), median (IQR)	57 (46–67)	0.207	–	–	58 (45–67)	0.146	–	–
Clinical stage III	59 (17.6)	0.001	3.03 (1.43–6.43)	0.004	37 (25.2)	0.008	1.23 (0.20–7.44)	0.825
Non-luminal subtypes	125 (37.3)	0.434	–	–	50 (34.0)	0.306	–	–
Ki-67 labeling index (per 10%), median (IQR)	31 (17–63)	<0.001	1.37 (1.18–1.58)	<0.001	27 (15–61)	0.048	1.39 (1.04–1.86)	0.025
Histological grade III	124 (37.0)	0.151	–	–	62 (42.2)	0.211	–	–
Pathological tumor size (per 10 mm), median (IQR)	20 (10–28)	0.990	–	–	21 (13–29)	0.004	1.34 (1.04–1.72)	0.024
Number of pathological lymph node metastases ≥4	41 (12.2)	0.803	–	–	32 (21.8)	0.001	7.42 (1.24–44.46)	0.028
Lymphovascular invasion	151 (45.1)	0.184	–	–	74 (50.3)	0.037	2.88 (0.30–27.32)	0.356
Neoadjuvant and/or adjuvant chemotherapy	242 (72.2)	0.248	–	–	108 (73.5)	0.079	–	–
Radiation therapy	162 (48.4)	0.297	–	–	87 (59.2)	0.356	–	–

Values are *n* (%) for categorical variables and median (IQR) for continuous variables. ^a^ Log-rank test for categorical variables; likelihood-ratio test for continuous variables. ^b^ Variables with univariate *p* < 0.05 were entered into the multivariable Cox model; *p* values by Wald test. A dash (–) indicates that the variable was not included in the multivariable model. ^c^ Interpret with caution owing to the small number of events. Abbreviations: HR, hazard ratio; CI, confidence interval; IQR, interquartile range.

**Table 3:** Association between blood group B and breast cancer-specific survival according to pathological nodal status and test for interaction.

No. of Metastatic Lymph Nodes	Blood Group	No. of Patients	No. of BCSS Deaths (%)	5-Year BCSS, % (95% CI)	10-Year BCSS, % (95% CI)	Adjusted HR (95% CI)	*p* Value	*p* for Interaction
0–3	Non-B	1003	47 (4.7)	97.7 (96.7–98.6)	94.1 (92.3–95.9)	1.00 (Reference)	—	<0.001 ^a^ (permutation-adjusted *p* = 0.004 ^b^)
	B	294	25 (8.5)	93.3 (90.4–96.3)	90.1 (86.3–94.0)	2.00 (1.23–3.26)	0.005	
≥4	Non-B	202	41 (20.3)	84.0 (78.9–89.3)	78.4 (72.6–84.6)	1.00 (Reference)	—	
	B	41	4 (9.8)	91.9 (83.5–100.0)	88.6 (78.6–99.8)	0.36 (0.13–1.00) ^c^	0.051	

BCSS rates were estimated using the Kaplan–Meier method. Adjusted HRs were estimated using a multivariable Cox proportional hazards model including blood group B, nodal status (0–3 vs. ≥4), their interaction term, age (per 10 years), clinical stage (0–II vs. III), breast cancer subtype (luminal vs. non-luminal), Ki-67 labeling index (per 10%), histological grade (I–II vs. III), pathological tumor size (per 10 mm), lymphovascular invasion (absent vs. present), neoadjuvant and/or adjuvant chemotherapy (no vs. yes), and radiation therapy (no vs. yes); *p* values were obtained using Wald tests. ^a^ Likelihood-ratio test for the interaction term. ^b^ Obtained from 1000 permutations of ABO blood group labels. ^c^ Based on only four events; the estimate should be interpreted with caution. Abbreviations: BCSS, breast cancer-specific survival; HR, hazard ratio; CI, confidence interval.

## Data Availability

The datasets generated during and/or analyzed during the current study are not publicly available because of patient privacy considerations but are available from the corresponding author on reasonable request.

## Supplementary Materials

The following supporting information can be downloaded at: https://www.mdpi.com/article/10.3390/cancers18213448/s1: Table S1: Univariate and multivariate analyses of prognostic factors for breast cancer-specific survival in the overall stage II–III cohort; Table S2: Overview of analyses performed, in the order conducted; Table S3: Interaction analyses and assessment of the proportional hazards assumption; Table S4: Sensitivity analyses of the association between blood group B and breast cancer-specific survival according to pathological nodal status; Table S5: Univariate and multivariate analyses of prognostic factors for breast cancer-specific survival in patients with stage II–III breast cancer and 0–3 pathological lymph node metastases; Table S6: Comparison of clinicopathological characteristics between blood group B and non-B patients with stage II–III breast cancer and 0–3 pathological lymph node metastases; Table S7: Breast cancer-specific deaths according to treatment sequence, pathological response, and blood group; Figure S1: Breast cancer-specific survival according to ABO blood group in patients with stage II–III breast cancer; Figure S2: Breast cancer-specific survival according to ABO blood group in patients with stage II–III breast cancer and ≥4 pathological lymph node metastases; Figure S3: Cumulative incidence of recurrence by pattern in patients with stage II–III breast cancer and 0–3 lymph node metastases: comparison between blood group B and non-B blood groups; Figure S4: Breast cancer-specific survival according to blood group (B vs. non-B) in patients with 0–3 pathological lymph node metastases, stratified by treatment sequence.
