# Supplementary material for: Prognostic Significance of Nodal Burden According to ABO Blood Group in Stage II–III Breast Cancer: An Exploratory Analysis
Source: Cancers (Basel). 2026 Oct 25;18(21):3448. doi: 10.3390/cancers18213448 (PMC13762008; doi:10.3390/cancers18213448)
Supplement: Supplementary file 1 [file cancers-18-03448-s001.zip › Table S1.pdf]

Table S1. Univariate and multivariate analyses of prognostic factors for breast cancer-specific survival in the overall stage II–III cohort.

Variables	Overall cohort (n=1540; 117 events)			
	N (%)	Univariate[a]	Multivariate[b]	
		P value	HR (95% CI)	P value
Age (per 10 years), median (IQR)	57 (47–68)	0.185	–	–
ABO blood group		0.641		0.283[c]
A	595 (38.6)		1.00 (Reference)	–
O	463 (30.1)		0.88 (0.56–1.37)	0.567
B	335 (21.8)		1.08 (0.67–1.73)	0.750
AB	147 (9.5)		0.51 (0.24–1.09)	0.083
Clinical stage III	276 (17.9)	**<0.001**	**1.87 (1.23–2.84)**	**0.003**
Non-luminal subtypes	561 (36.4)	**0.047**	1.20 (0.78–1.85)	0.402
Ki-67 labeling index (per 10%), median (IQR)	30 (15–60)	**<0.001**	**1.34 (1.22–1.47)**	**<0.001**
Histological grade III	553 (35.9)	**0.003**	0.68 (0.41–1.11)	0.119
Pathological tumor size (per 10 mm), median (IQR)	21 (11–28)	**<0.001**	**1.14 (1.06–1.23)**	**<0.001**
Number of pathological lymph node metastases ≥4	243 (15.8)	**<0.001**	**2.82 (1.77–4.50)**	**<0.001**
Lymphovascular invasion	704 (45.7)	**<0.001**	**2.23 (1.46–3.41)**	**<0.001**
Neoadjuvant and/or adjuvant chemotherapy	1091 (70.8)	**<0.001**	1.22 (0.68–2.17)	0.502
Radiation therapy	836 (54.3)	**0.004**	0.93 (0.59–1.48)	0.772

Values are n (%) for categorical variables and median (IQR) for continuous variables. [a]Log-rank test for categorical variables; likelihood-ratio test for continuous variables. [b]Variables with univariate $P < 0.05$ were entered into the multivariable Cox model; ABO blood group was entered regardless of its univariate P value, with group A as the reference. P values by Wald test. [c]Overall Wald test for ABO blood group. A dash (–) indicates that the variable was not included in the multivariable model or not applicable to the reference category. Abbreviations: HR, hazard ratio; CI, confidence interval; IQR, interquartile range.
