# Supplementary material for: Prognostic Significance of Nodal Burden According to ABO Blood Group in Stage II–III Breast Cancer: An Exploratory Analysis
Source: Cancers (Basel). 2026 Oct 25;18(21):3448. doi: 10.3390/cancers18213448 (PMC13762008; doi:10.3390/cancers18213448)
Supplement: Supplementary file 1 [file cancers-18-03448-s001.zip › Table S2.pdf]

Table S2. Overview of analyses performed, in the order conducted.

Step	Analysis	Population	Presented in	Timing
1	Clinicopathological characteristics by ABO blood group	All (n = 1540)	Table 1	Prespecified
2	BCSS by ABO blood group	All	Figure S1; Table S1	Prespecified
3	Prognostic factor analyses stratified by ABO blood group (4 blood groups × 10 factors)	All	Table 2; Figure 1	Prespecified (primary aim)
4	Interaction between blood group B and nodal status (0–3 vs. ≥4); single formal test	All	Table 3	Post hoc, based on step 3 and the established ≥4-node threshold
5	Permutation test across the four blood groups; sensitivity analyses; bootstrap resampling to assess internal stability; proportional hazards assessment	All	Tables 3, S3, and S4	Added in revision
6	Blood group–specific associations of continuous factors (nodal burden, tumor size, Ki-67) with heterogeneity across the four ABO blood groups; interactions between blood group B and these factors	All	Figure 2; Table S3	Added in revision (exploratory)
7	Characteristics, BCSS, prognostic factors, and recurrence patterns: blood group B vs. non-B	0–3 nodes (n = 1297)	Figure 3; Tables S5 and S6; Figure S3	Post hoc (exploratory)
8	BCSS by ABO blood group	≥4 nodes (n = 243)	Figure S2	Post hoc (exploratory)
9	Analyses stratified by neoadjuvant chemotherapy, ypN status, and clinical stage	All; 0–3 nodes	Figure S4; Table S7	Post hoc; added in revision

No adjustment for multiple testing was applied except for the permutation-based adjustment of the interaction test (step 5). All analyses other than those in steps 1–3 should be regarded as exploratory. Abbreviation: BCSS, breast cancer-specific survival.
