# Supplementary material for: Prognostic Significance of Nodal Burden According to ABO Blood Group in Stage II–III Breast Cancer: An Exploratory Analysis
Source: Cancers (Basel). 2026 Oct 25;18(21):3448. doi: 10.3390/cancers18213448 (PMC13762008; doi:10.3390/cancers18213448)
Supplement: Supplementary file 1 [file cancers-18-03448-s001.zip › Table S3.pdf]

Table S3. Interaction analyses and assessment of the proportional hazards assumption.

(A) Interaction between each ABO blood group (vs. all others) and pathological nodal status (0–3 vs. ≥4)

Blood group	Adjusted HR, 0–3 nodes	Adjusted HR, ≥4 nodes	P for interaction
A	0.79	2.06	**0.015**
O	0.95	0.88	0.850
B	2.00	0.36	**<0.001**
AB	0.32	0.84	0.198

Permutation-adjusted P for the smallest observed interaction P value (blood group B): 0.004 (1,000 permutations of ABO blood group labels). Each model was adjusted for the covariates listed in Table 3.

(B) Interaction between blood group B and continuous prognostic factors

Factor	HR in non-B (95% CI)	HR in blood group B (95% CI)	P for interaction
Nodal burden (per doubling of number of metastatic nodes + 1)	1.79 (1.49–2.14)	0.98 (0.70–1.35)	**<0.001**
Pathological tumor size (per 10 mm)	1.17 (1.09–1.27)	0.96 (0.76–1.21)	0.074
Ki-67 labeling index (per 10%)	1.30 (1.17–1.44)	1.47 (1.26–1.72)	0.123

Each model included blood group B, the factor, their interaction term, and the remaining covariates listed in Table 3 (with nodal status omitted from the nodal burden model). Exploratory analyses without adjustment for multiple testing. Estimates differ slightly from those in Figure 2, in which factor-specific terms were estimated separately for each of the four ABO blood groups and heterogeneity was tested across all four groups.

(C) Proportional hazards assessment for the primary interaction model (Schoenfeld residuals)

Term	Chi-square	P value
Blood group B	1.66	0.197
≥4 lymph node metastases	0.31	0.576
Age (per 10 years)	0.59	0.441
Clinical stage III	0.00	0.966
Non-luminal subtypes	**9.97**	**0.002**
Ki-67 labeling index (per 10%)	**25.78**	**<0.001**
Histological grade III	**15.97**	**<0.001**
Pathological tumor size (per 10 mm)	1.07	0.300
Lymphovascular invasion	0.02	0.901
Neoadjuvant and/or adjuvant chemotherapy	0.27	0.604
Radiation therapy	1.51	0.219
Blood group B × ≥4 lymph node metastases	0.68	0.411
Global test	**42.68**	**<0.001**

The proportional hazards assumption was violated for non-luminal subtype, Ki-67 labeling index, and histological grade, but not for blood group B, nodal status, or their interaction term. A sensitivity analysis using time-varying coefficients for these three covariates yielded consistent results (Table S4). Abbreviations: HR, hazard ratio; CI, confidence interval.
