# Supplementary material for: Prognostic Significance of Nodal Burden According to ABO Blood Group in Stage II–III Breast Cancer: An Exploratory Analysis
Source: Cancers (Basel). 2026 Oct 25;18(21):3448. doi: 10.3390/cancers18213448 (PMC13762008; doi:10.3390/cancers18213448)
Supplement: Supplementary file 1 [file cancers-18-03448-s001.zip › Table S4.pdf]

Table S4. Sensitivity analyses of the association between blood group B and breast cancer-specific survival according to pathological nodal status.

Analysis	HR for blood group B, 0–3 nodes (95% CI)	HR for blood group B, ≥4 nodes (95% CI)	P for interaction
Primary analysis	2.00 (1.23–3.26)	0.36 (0.13–1.00)	<0.001
Firth penalized Cox regression	2.02 (1.24–3.29)	0.40 (0.15–1.07)	<0.001
Unadjusted model	1.92 (1.18–3.13)	0.45 (0.16–1.26)	0.005
Parsimonious model (clinical stage and Ki-67 index)	1.96 (1.21–3.18)	0.39 (0.14–1.08)	0.002
Chemotherapy categorized as none, adjuvant, or neoadjuvant	2.01 (1.23–3.27)	0.32 (0.11–0.90)	<0.001
Dichotomized covariates: standard ROC cutoffs (age 66 y, Ki-67 55%, size 28 mm)	1.93 (1.18–3.14)	0.41 (0.15–1.15)	0.003
Dichotomized covariates: 5-year time-dependent ROC cutoffs (age 50 y, Ki-67 55%, size 25 mm)	2.03 (1.24–3.31)	0.43 (0.15–1.19)	0.002
Dichotomized covariates: conventional cutoffs (age 50 y, Ki-67 20%, size 20 mm)	1.94 (1.19–3.16)	0.45 (0.16–1.27)	0.005
Excluding Rh-negative patients (n = 13)	1.97 (1.21–3.20)	0.39 (0.14–1.09)	0.002
Time-varying coefficients for covariates violating the proportional hazards assumption (Ki-67, grade, subtype)	2.02 (1.24–3.28)	0.34 (0.12–0.96)	<0.001
Bootstrap resampling (1,000 replicates; median and 2.5th–97.5th percentiles)	2.03 (1.18–3.22)	0.35 (0.10–0.80)	–

HRs compare blood group B with non-B blood groups within each nodal stratum. Unless otherwise stated, models were adjusted for the covariates listed in Table 3. P for interaction was obtained using likelihood-ratio tests (penalized likelihood-ratio test for Firth regression).

Dichotomized cutoffs were derived from receiver operating characteristic analysis without consideration of follow-up time, from time-dependent receiver operating characteristic analysis at 5 years using inverse probability of censoring weighting, or from commonly used thresholds. Proportional hazards were assessed using Schoenfeld residuals (Table S3); covariates violating the assumption were modeled with time-varying coefficients (covariate × log time).

Bootstrap estimates are medians with 2.5th–97.5th percentiles of 1,000 resamples; 11 resamples in which the model did not converge (no BCSS deaths among patients with blood group B and ≥4 nodes) were excluded. The interaction was significant ($P < 0.05$) in 92.6% of the 989 converged resamples. Permutation-adjusted P for interaction, accounting for the selection of blood group B among four ABO blood groups: 0.004.

Abbreviations: HR, hazard ratio; CI, confidence interval; ROC, receiver operating characteristic.
