# Supplementary material for: Prognostic Significance of Nodal Burden According to ABO Blood Group in Stage II–III Breast Cancer: An Exploratory Analysis
Source: Cancers (Basel). 2026 Oct 25;18(21):3448. doi: 10.3390/cancers18213448 (PMC13762008; doi:10.3390/cancers18213448)
Supplement: Supplementary file 1 [file cancers-18-03448-s001.zip › Table S5.pdf]

Table S5. Univariate and multivariate analyses of prognostic factors for breast cancer-specific survival in patients with stage II–III breast cancer and 0–3 pathological lymph node metastases

Variables	Patients with 0–3 pathological lymph node metastases(n=1297; 72 events)			
	N (%)	Univariate[a]	Multivariate	
		P value	HR (95% CI)[b]	P value
Age (per 10 years), median (IQR)	58 (47–68)	0.106	–	–
Blood group B	294 (22.7)	**0.007**	**2.04 (1.25–3.32)**	**0.004**
Clinical stage III	180 (13.9)	**<0.001**	**2.66 (1.60–4.42)**	**<0.001**
Non-luminal subtypes	497 (38.3)	0.065	–	–
Ki-67 labeling index (per 10%), median (IQR)	30 (15–60)	**<0.001**	**1.30 (1.15–1.47)**	**<0.001**
Histological grade III	487 (37.5)	**0.008**	0.74 (0.39–1.41)	0.354
Pathological tumor size (per 10 mm), median (IQR)	20 (10–27)	**<0.001**	**1.20 (1.09–1.31)**	**<0.001**
Number of pathological lymph node metastases ≥2	241 (18.6)	0.108	–	–
Lymphovascular invasion	534 (41.2)	**0.020**	**1.91 (1.16–3.14)**	**0.011**
Neoadjuvant and/or adjuvant chemotherapy	875 (67.5)	**0.023**	0.97 (0.51–1.85)	0.938
Radiation therapy	600 (46.3)	0.440	–	–

Values are n (%) for categorical variables and median (IQR) for continuous variables. [a]Log-rank test for categorical variables; likelihood-ratio test for continuous variables. [b]Variables with univariate $P < 0.05$ were entered into the multivariable Cox model; P values by Wald test. A dash (–) indicates that the variable was not included in the multivariable model.

Abbreviations: HR, hazard ratio; CI, confidence interval; IQR, interquartile range.
