# Supplementary material for: Prognostic Significance of Nodal Burden According to ABO Blood Group in Stage II–III Breast Cancer: An Exploratory Analysis
Source: Cancers (Basel). 2026 Oct 25;18(21):3448. doi: 10.3390/cancers18213448 (PMC13762008; doi:10.3390/cancers18213448)
Supplement: Supplementary file 1 [file cancers-18-03448-s001.zip › Table S6.pdf]

Table S6. Comparison of clinicopathological characteristics between blood group B and non–B patients with stage II–III breast cancer and 0–3 pathological lymph node metastases

Variables	Blood group		P value
	Non-B (n=1003)	B (n=294)	
Age (years), median (IQR)	58 (47–68)	57 (46–67)	0.449
Gender (Female)	999 (99.6)	293 (99.7)	1.000
Performance status (ECOG ≥ 1)	87 (8.7)	33 (11.2)	0.208
Clinical T stage (cT3–4)	105 (10.5)	31 (10.5)	1.000
Clinical lymph node status			0.353
cN0	643 (64.1)	179 (60.9)	
cN1	294 (29.3)	89 (30.3)	
cN2–3	66 (6.6)	26 (8.8)	
Clinical stage at diagnosis			0.749
0	16 (1.6)	6 (2.0)	
I	179 (17.8)	53 (18.0)	
II	673 (67.1)	190 (64.6)	
III	135 (13.5)	45 (15.3)	
Subtypes			0.642
Luminal	621 (61.9)	179 (60.9)	
Luminal-HER2	125 (12.5)	35 (11.9)	
HER2	88 (8.8)	33 (11.2)	
Triple negative	169 (16.8)	47 (16.0)	
Ki-67 labeling index (%), median (IQR)	30 (15–60)	32 (17–63)	0.376
Histological grade			
Grade I	143 (14.3)	49 (16.7)	0.444
Grade II	486 (48.5)	132 (44.9)	
Grade III	374 (37.3)	113 (38.4)	
Histological type			0.971
Invasive ductal carcinoma	849 (84.6)	251 (85.4)	
Invasive lobular carcinoma	75 (7.5)	21 (7.1)	
Others	79 (7.9)	22 (7.5)	
Pathological tumor size (mm), median (IQR)	20 (10–27)	19 (9–25)	0.311
Number of pathological lymph node metastases ≥1	505 (50.4)	139 (47.3)	0.389
Number of pathological lymph node metastases ≥2	199 (19.8)	42 (14.3)	**0.033**
Lymphovascular invasion	414 (41.3)	120 (40.8)	0.946
Pathological complete response rate (%)[a]	26.9 (106/394)	29.2 (35/120)	0.641
Surgical procedure			0.147
Mastectomy	631 (62.9)	199 (67.7)	
Breast-conserving surgery (BCS)	372 (37.1)	95 (32.3)	
Axillary surgery			0.546
Sentinel lymph node biopsy (SLNB) only	427 (42.6)	119 (40.5)	

Axillary lymph node dissection (ALND)	576 (57.4)	175 (59.5)	
Radiation therapy			0.173
Breast irradiation alone	331 (33.0)	81 (27.6)	
Breast and regional irradiation[b]	146 (14.6)	42 (14.3)	
No irradiation	526 (52.4)	171 (58.2)	
Endocrine therapy	705 (70.3)	204 (69.4)	0.772
Anti-HER2 therapy	211 (21.0)	60 (20.4)	0.871
Neoadjuvant chemotherapy	394 (39.3)	120 (40.8)	0.636
Adjuvant chemotherapy	299 (29.8)	100 (34.0)	0.173
Chemotherapy regimen			0.198
Anthracycline- and taxane-containing regimens	483 (48.2)	153 (52.0)	
Taxane-containing regimens without anthracycline	84 (8.4)	33 (11.2)	
Anthracycline-containing regimens without taxane	94 (9.4)	20 (6.8)	
Others	6 (0.6)	2 (0.7)	
No chemotherapy	336 (33.5)	86 (29.3)	
Completion rate of intravenous chemotherapy (%)[c]	90.1 (597/663)	87.9 (181/206)	0.364

Values are expressed as medians (interquartile ranges) or n (%). P values represent comparisons between the B and non-B groups and were calculated using the Wilcoxon rank-sum test for continuous variables and FisherÄs exact test or the chi-square test for categorical variables, as appropriate.

[a]Pathological complete response (pCR) was defined as ypT0/is ypN0 and was assessed among patients who received neoadjuvant chemotherapy (n=514). Values are presented as percentages, with the number of patients achieving pCR/ number of patients receiving neoadjuvant chemotherapy shown in parentheses.

[b]Breast and regional irradiation includes postmastectomy radiation therapy (PMRT).

[c]The completion rate of intravenous chemotherapy was evaluated in 869 patients who received the treatment. Values are presented as percentages, with the number of patients completing intravenous chemotherapy/ number of patients receiving intravenous chemotherapy shown in parentheses.

Abbreviations: IQR, interquartile range; ECOG, Eastern Cooperative Oncology Group; HER2, human epidermal growth factor receptor 2.
