# Supplementary material for: Prognostic Significance of Nodal Burden According to ABO Blood Group in Stage II–III Breast Cancer: An Exploratory Analysis
Source: Cancers (Basel). 2026 Oct 25;18(21):3448. doi: 10.3390/cancers18213448 (PMC13762008; doi:10.3390/cancers18213448)
Supplement: Supplementary file 1 [file cancers-18-03448-s001.zip › Table S7.pdf]

Table S7. Breast cancer–specific deaths according to treatment sequence, pathological response, and blood group.

(A) Treatment sequence and pathological nodal status

Treatment sequence	0–3 nodes: Non-B	0–3 nodes: B	≥4 nodes: Non-B	≥4 nodes: B
Upfront surgery (pN)	20/609 (3.3)	6/174 (3.4)	14/105 (13.3)	1/21 (4.8)
Neoadjuvant chemotherapy (ypN)	27/394 (6.9)	19/120 (15.8)	27/97 (27.8)	3/20 (15.0)

Values are BCSS deaths/patients (%). Adjusted HR for blood group B among patients with 0–3 nodes: upfront surgery, 0.97 (95% CI, 0.38–2.46; P = 0.942); neoadjuvant chemotherapy, 3.00 (95% CI, 1.63–5.53; P < 0.001); P for interaction between blood group B and neoadjuvant chemotherapy = 0.024 (adjusted for the covariates listed in Table 3 except chemotherapy). Three-way interaction (blood group B × nodal status × neoadjuvant chemotherapy) in the overall cohort: P = 0.553.

(B) Pathological response among patients who received neoadjuvant chemotherapy

Pathological response	Non-B	B	Unadjusted HR for B (95% CI)
Pathological complete response (ypT0/is ypN0)	0/106 (0)	3/35 (8.6)	–[a]
ypN0 with residual invasive disease in the breast	10/156 (6.4)	7/42 (16.7)	–
All ypN0[b]	10/263 (3.8)	10/78 (12.8)	**3.66 (1.52–8.79)**
ypN1–3	17/131 (13.0)	9/42 (21.4)	1.90 (0.85–4.27)
ypN ≥4	27/97 (27.8)	3/20 (15.0)	–

Values are BCSS deaths/patients (%). Pathological complete response rate: non-B, 21.6% (106/491); B, 25.0% (35/140). [a]Not estimable because there were no events among non-B patients. [b]Includes two patients with missing breast response data. P for interaction between blood group B and ypN0 (vs. ypN1–3) = 0.33.

(C) Blood group B among patients with neoadjuvant chemotherapy and 0–3 nodes, by clinical stage

Clinical stage at diagnosis	Non-B	B	Unadjusted HR for B (95% CI)
0–II	15/280 (5.4)	10/81 (12.3)	**2.40 (1.08–5.35)**
III	12/114 (10.5)	9/39 (23.1)	**2.68 (1.13–6.38)**

Values are BCSS deaths/patients (%). P for interaction between blood group B and clinical stage III = 0.70. All analyses in this table are post hoc and exploratory, and event numbers are small. A dash (–) indicates not estimated. Bold values indicate P < 0.05. Abbreviations: BCSS, breast cancer–specific survival; HR, hazard ratio; CI, confidence interval.
